# Intracellular ROS Scavenging and Anti-Inflammatory Activities of *Oroxylum indicum* Kurz (L.) Extract in LPS plus IFN-*γ*-Activated RAW264.7 Macrophages

**DOI:** 10.1155/2020/7436920

**Published:** 2020-05-27

**Authors:** Benjawan Dunkhunthod, Chutima Talabnin, Mark Murphy, Kanjana Thumanu, Patcharawan Sittisart, Tanaporn Hengpratom, Griangsak Eumkeb

**Affiliations:** ^1^School of Preclinic, Institute of Science, Suranaree University of Technology, Nakhon Ratchasima 30000, Thailand; ^2^School of Chemistry, Institute of Science, Suranaree University of Technology, Nakhon Ratchasima 30000, Thailand; ^3^School of Biomolecular Science, Liverpool John Moores University, Liverpool L3 3AF, UK; ^4^Synchrotron Light Research Institute (Public Organization), Nakhon Ratchasima 30000, Thailand; ^5^Division of Environmental Science, Faculty of Liberal Arts and Science, Sisaket Rajabhat University, Sisaket 33000, Thailand

## Abstract

*Oroxylum indicum* (L.) Kurz has been used as plant-based food and herbal medicine in many Asian countries. The aim of the present study was to examine the antioxidant and anti-inflammatory activities of *O. indicum* extract (*O. indicum*) in RAW264.7 cells activated by LPS plus IFN-*γ*. The phytochemical compounds in *O. indicum* were identified by GC-MS and LC-MS/MS. Five flavonoids (luteolin, apigenin, baicalein, oroxylin A, and quercetin) and 27 volatile compounds were found in *O. indicum*. *O. indicum* presented antioxidant activities, including reducing ability by FRAP assay and free radical scavenging activity by DPPH assay. Moreover, *O. indicum* also suppressed LPS plus IFN-*γ*-activated reactive oxygen species generation in RAW264.7 macrophages. It possessed the potent anti-inflammatory action through suppressing nitric oxide (NO) and IL-6 secretion, possibly due to its ability to scavenge intracellular ROS. The synchrotron radiation-based Fourier transform infrared (SR-FTIR) spectroscopy results showed the alteration of signal intensity and integrated areas relating to lipid and protein of the activated RAW264.7 macrophages compared to unactivated cells. This is the first report of an application of the SR-FTIR technique to evaluate biomolecular changes in activated RAW264.7 cells. Our results indicate that *O. indicum* may be used as a potential source of nutraceutical for the development of health food supplement or a novel anti-inflammatory herbal medicine.

## 1. Introduction

Inflammation is a response of the immune system to injury, irritation, or infection caused by invading pathogens, radiation exposure, very high or low temperatures, or autoimmune processes. Lipopolysaccharide (LPS), an outer membrane component of Gram-negative bacteria, has been reported as one of the major causes of septic shock [1]. In response to endotoxin stimulation, a variety of immune cells can be activated. In the innate immunity system, macrophages play pivotal roles in the cellular host's defense against infection and tissue injury [2]. Inflammation is considered to be beneficial when it is short term and under control within the immune system (acute inflammation). However, if the inflammatory process has been going on for too long (chronic inflammation) or if the inflammatory response occurs in places where it is not needed, it can become problematic.

During inflammation responses, mast cells, monocytes, macrophages, lymphocytes, and other immune cells are first activated. The cells are recruited to the site of damage, resulting in the generation of reactive oxygen species that damage macromolecules, including DNA. At the same time, these inflammatory cells also produce large amounts of inflammatory mediators such as metabolites of arachidonic acid, nitric oxide (NO), proinflammatory cytokines, chemokines, prostaglandins, inducible enzymes, and growth factors [3]. Excessive production of intracellular ROS can cause oxidative stress associated with redox unbalance [4]. This imbalance leads to damage of essential biomolecules and cells, with potential impact on the whole organism. Accordingly, excessive oxidative stress and chronic inflammation can cause chronic diseases such as cancer, aging, diabetes, obesity, cardiovascular diseases, Alzheimer's, and Parkinson's disease. Therefore, oxidative stress and inflammation must be adequately controlled to prevent the progressions of chronic diseases.

Over the past few decades, studies have investigated the possible protective role of plant foods against chronic diseases. Several epidemiological studies have revealed that higher consumption of fruits and vegetables is associated with a lower risk of chronic diseases [5]. The usage of herbs or traditional herbal medicines that are complementary and alternative medicines for the management of inflammation has increased because of the concerns about the adverse side effects of nonsteroidal anti-inflammatory drugs [6]. These indigenous vegetables can be readily available in the local area. Consequently, the consumption of these vegetables would be appropriate to control and also reduce the cost of inflammatory management.

*Oroxylum indicum* (L.) Kurz is a species of flowering plant belonging to the Bignoniaceae family. Fruit pods of this plant have been used in many Asian countries as a plant-based food and herbal medicine for thousands of years without any known adverse effects [7–9]. Numerous scientific studies showed that *O. indicum* was not toxic even in experimental animals, even up to high doses [10–12]. The fruit pods of this plant are rich in nutrients concerning dietary fiber, essential amino acids, minerals, and fatty acids [8]. Every part of this tree is also an essential source of several medicinally essential flavonoids, including baicalein, chrysin, and oroxylin A, which have contributed to many of the biological activities. The crude extracts and its isolated compounds exhibit a broad spectrum of *in vitro* and *in vivo* pharmacological activities involving antioxidant, antidiabetic, anti-adipogenesis, hepatoprotective, and anti-inflammatory activities [7, 11, 13, 14].

Currently, Fourier transform infrared (FTIR) microspectroscopy is widely used as a tool to monitor the changes in various biological samples such as bacteria, apoptotic and necrotic cell death, stem cell differentiation, and adipocyte cells [14–17]. FTIR technique is based on the absorption of infrared (IR) light of chemical bonds by vibrational transitions. This is a label-free and nondestructive technique that enables the analysis of biological samples with no need for staining and without sample destruction [18]. However, infrared microspectroscopy of cellular samples is not a single-molecule detection technique because of the limited spatial resolution when working on individual cells and the lack of sensitivity of the detectors while analyzing thick tissue sections [19]. In this context, the use of synchrotron radiation-based Fourier transform infrared (SR-FTIR) microspectroscopy was considered to explore the molecular chemistry within microstructures of individual single cells with a high signal [20]. To date, there are no studies available for SR-FTIR microspectroscopy to detect molecular changes in LPS plus IFN-*γ*-activated RAW264.7 cells. There is considerable interest in applying SR-FTIR microspectroscopy to identify biochemical changes in LPS plus IFN-*γ*-activated RAW264.7 cells.

Therefore, this study aimed to investigate the antioxidant and anti-inflammatory activities of *O. indicum* in RAW264.7 cells activated by LPS plus IFN-*γ*. We also examined the biochemical alteration in LPS plus IFN-*γ*-activated RAW264.7 cells upon treatment with *O. indicum* by using SR-FTIR microspectroscopy.

## 2. Materials and Methods

### 2.1. Chemicals and Reagents

Vitamin C (VIT.C) was purchased from Fluka Chemie GmbH (Buchs, Switzerland). 2,2-Diphenyl-1-picryl-hydrazyl (DPPH), sodium nitrite, LPS (Escherichia coli; O111 : B4), 2′,7′-dichlorofluorescein-diacetate (DCFH-DA), and N-acetyl-cysteine (NAC) were purchased from Sigma-Aldrich (St. Louis, USA). Dimethyl sulfoxide (DMSO) was bought from Amresco Inc. (Solon, USA). 6-Hydroxy-2,5,7,8-tetramethylchroman-2-carboxylic acid (Trolox) was obtained from Sigma-Aldrich Chemie GmbH (Steinheim, Germany). Dexamethasone (DEX) was obtained from G Bioscience (St. Louis, USA). 3-(4,5-Dimethylthiazol-2-yl)-2,5-diphenyl-tetrazolium bromide (MTT), Roswell Park Memorial Institute (RPMI) 1640, fetal bovine serum (FBS), penicillin-streptomycin, and N-2-hydroxyethylpiperazine-N-2-ethane sulfonic acid (HEPES) were obtained from Gibco Invitrogen (Grand Island, NY, USA). Griess-Ilosvay's reagent was purchased from Merck KGaA (Darmstadt, Germany). Mouse interferon-gamma (mIFN-*γ*) was purchased from Pierce Protein Research Products (Rockford, USA). TMB substrate: 3,3′,5,5′ tetramethylbenzidine was purchased from PanReac AppliChem ITW Reagents (Darmstadt, Germany). Elisa kits for IL-6 and TNF-*α* were obtained from R&D Systems, Inc. (Minneapolis, USA). Baicalein and all other chemical standards for LC-MS/MS analysis were obtained from INDOFINE Chemical Company, Inc. (Hillsborough, NJ, USA). Other reagents used were all of analytical grade.

### 2.2. Preparation of Plant Extract

The fresh fruit pods of *O. indicum* were purchased from the local market at Wang Nam Khiao District, Nakhon Ratchasima province, Thailand. The plant samples were identified by a botanist at the Institute of Science, Suranaree University, Thailand, and the voucher specimens were kept at the flora of Suranaree University of Technology Herbarium (SOI0808U). Fresh fruit pods were washed and cut into small pieces and then dried in the oven at 40°C for 2 days. The dried pieces were pulverized using a mechanical grinder. *O. indicum* dry powder (500 g) was extracted with 95% ethanol by a soxhlation for 8 h and then filtered through Whatman filter paper. The ethanolic extract was concentrated and lyophilized to obtain the powder of *O. indicum*. The crude extracts were stored at −20°C till use in subsequent experiments. *O. indicum* was dissolved in 100% DMSO and diluted to 0.06% (v/v) in the cell culture medium.

### 2.3. GC-MS Analysis

The phytoconstituents present in the extract of *O. indicum* were analyzed by gas chromatography-mass spectrometry (GC-MS) using a Bruker 450-GC/Bruker 320-MS equipped with Rtx-5MS fused silica capillary column (30 m × 0.25 mm; 0.25 *μ*m in the thickness of film). Helium was used as the carrier gas with a flow rate of 1 mL min^−1^. The injector temperature was operated at 250°C, and the oven temperature was programmed from 110°C (2 min), 200°C (3 min), and 280°C (20 min) with rates of 0, 10, and 5°C min^−1^, respectively. The mass spectral data were taken with an electron energy of 70 eV. The ion source and transfer line temperature were kept at 200°C. The mass spectra were obtained by a centroid scan of the mass range from 45 to 500 atomic mass units. Interpretation of the mass spectrum of GC-MS was made by using NIST mass spectral library 2008.

### 2.4. Liquid Chromatography-Mass Spectrometer (LC-MS/MS) Quantification of the Selected Phenols and Flavonoids

The analyses were performed on the Dionex Ultimate 3000 UHPLC system (Dionex, USA) coupled with electrospray ionization (ESI) tandem mass spectrometer (micro-TOF-Q II) (Bruker, Germany). The injection volume of all samples was 5 *μ*L. The separation was achieved using a Zorbax SB-C18 (250  mm × 4.6  mm × 3.5 *μ*m (Agilent Technologies, USA)) and thermostat at 35°C, with a flow rate of 0.8 mL min^−1^ of the mobile phase, which included deionized water containing 0.1% formic acid (FA) as solvent A and acetonitrile containing 0.1% formic acid as solvent B. The gradient elution was performed using the following solvent gradient: starting with 30% B, reaching 80% B at 30 min, and holding until 38 min, reducing to 30% B in 2 min and holding until the run ending at 45 min. Detection of eluted components, ionized by ESI, was performed in the mass scanning mode in the range of 50 *m*/*z* to 1,500 *m*/*z* at negative ion polarity. The nebulizer gas (N_2_) was 2 Bar, drying gas was 8 L min^−1^, the dryer heater temperature was 180°C, and the capillary voltage was 4.5 kV. The LC-QTOF data were collected and processed by Compass 1.3 software (Bruker, Germany). The target phenolic and flavonoid compounds were identified and quantified with Bruker Quant Analysis Version 2.0 SP 5 software.

The calibration curves were constructed from peak areas of different concentrations of the reference standard (from 0.5 *μ*g mL^−1^ to 250 *μ*g mL^−1^), and the concentration of targeted compounds was calculated based on the equation for linear regression obtained from the calibration curves.

### 2.5. Ferric-Reducing/Antioxidant Power (FRAP) Assay

FRAP is based on the detection of the sample capacity to reduce ferric ions, which is measured as a change in the absorbance of the ferrous TPTZ complex. The assay was carried out, according to Rupasinghe et al. [21]. Briefly, the working FRAP reagent was prepared by mixing 300 mM acetate buffer (pH 3.6), a solution of 10 mM 2,4,6-tripyridyl-s-triazine (TPTZ) in 10 mM hydrochloric acid and 20 mM ferric chloride at 10 : 1:1 (v/v/v). The working FRAP reagent (180 *μ*L) and sample solutions (20 *μ*L) were mixed in a 96-well plate for 6 min. The absorbance was measured at 595 nm by using a microplate reader (Bio-Rad Laboratories, Inc., USA). The standard calibration curve was developed using different concentrations of Trolox and VIT.C. FRAP values were expressed as a milligram of Trolox equivalent antioxidant capacity (TREA) or ascorbic equivalent antioxidant capacity (VCEA) per gram of dry extract.

### 2.6. DPPH Radical Scavenging Activity Assay

The total free radical scavenging capacity of *O. indicum* was estimated according to the method of Yang et al. [22]. Briefly, one hundred microliters of the sample at different concentrations were added to 100 *μ*L of DPPH solution (0.2 mM) in a 96-well plate. The mixture was shaken vigorously at room temperature for 15 min in the dark and measured the absorbance at 517 nm by using a microplate reader (Bio-Rad Laboratories, Inc, USA). Trolox and VIT.C were used as a positive control. The free radical scavenging activity was calculated as follows:

(1) $$\begin{array}{c} \text{scavenging rate}\left(\%\right)=\left[\frac{A_{\text{control}}-A_{\text{sample}}}{A_{\text{control}}}\right]\times 100. \end{array}$$

The IC_50_ of DPPH˙ was determined from a dose-response curve using linear regression analysis. Decreasing DPPH solution absorption indicates an increase of DPPH radical scavenging activity.

### 2.7. Cell Culture

The RAW264.7 macrophage cells (Cell Lines Service, Eppelheim, Germany) were cultured at 37°C, 5% CO_2_ in RPMI-1640 medium supplemented with 10% heat-inactivated FBS and 100 U mL^−1^ penicillin-streptomycin.

### 2.8. *In Vitro* Cytotoxic Test (MTT Assay)

The cytotoxic effect of *O. indicum* on cell viability was determined by using a tetrazolium dye (MTT) colorimetric assay [23]. Briefly, the cells were seeded in a 96-well plate at a density of 2 × 10^4^ cells/well and allowed to adhere for 24 h. Cells were treated with different concentrations of *O. indicum* for 24 h. After incubation, the culture medium was removed, and 0.5 mg mL^−1^ (final concentration) MTT was added. Then, the cells were further incubated for 4 h at 37°C. Formazan crystal formed by viable cells was dissolved in DMSO and absorbance was measured at 540 nm with a microplate spectrophotometer (Bio-Rad Laboratories, Inc., USA).

### 2.9. Assessment of Intracellular ROS Scavenging Activity

The intracellular ROS scavenging capacity of *O. indicum* in RAW264.7 cells induced by LPS plus IFN-*γ* was measured using a DCFH-DA fluorescent probe, according to the method described by Sittisart and Chitsomboon [23]. Briefly, RAW264.7 cells were seeded in a 96-well black plate at 2.0 × 10^4^ cells/well and incubated overnight. Then, the culture medium was removed, and the cells were pretreated with *O. indicum* at the concentration of 50, 100, or 200 *μ*g mL^−1^, VIT.C 50 *μ*g mL^−1^, baicalein 5 *μ*g mL^−1^, or a selective ROS scavenger, NAC, for 3 h. Then, the cells were activated with 1 *μ*g mL^−1^ LPS plus 10 ng mL^−1^ IFN-*γ* and another 24-hour incubation. After removing the medium, the cells were treated with 20 M DCFH-DA in Hank's Balanced Salt Solution (HBSS) for 30 min and then washed with PBS twice. The fluorescence intensity was measured using a Gemini EM fluorescence microplate reader (Molecular Devices, Sunnyvale, CA) with an excitation wavelength of 485 nm and an emission wavelength of 535 nm. The percentage of DCF fluorescence intensity was calculated by the following formula: DCF fluorescence intensity (%) = (DCF fluorescence intensity_test group_/DCF fluorescence intensity_control group_) × 100. The IC_35,_ IC_40_, and IC_50_ of *O. indicum* were also calculated from a dose-response curve using linear regression analysis.

### 2.10. Determination of Nitric Oxide (NO) and Proinflammatory Cytokines (IL-6 and TNF-*α*) Production

The anti-inflammatory activities of *O. indicum* were evaluated by measuring the level of NO and proinflammatory cytokines (IL-6 and TNF-*α*) production in LPS plus IFN-*γ*-activated RAW264.7 cells. The cells were seeded at a density of 6 × 10^5^ cells/well in a 6-well plate and then incubated overnight. After incubation, the culture medium was removed, and the cells were pretreated with different concentrations of *O. indicum* (50, 100, and 200 *μ*g mL^−1^) or the anti-inflammatory agent, DEX (1 *μ*M), for 3 h. Then, the cells were activated with 1 *μ*g mL^−1^ LPS plus 10 ng mL^−1^ IFN-*γ* and incubated for 24 h. The supernatant was collected for further analysis of NO using Griess reagent and determined the level of TNF-*α* and IL-6 with the ELISA kits.

The level of NO in the culture media was detected as nitrite, a major stable product of NO, using Griess reagent as described by Sittisart et al. [24]. Briefly, 100 *μ*L of cell culture medium was mixed with an equal volume of Griess reagent in a 96-well plate and incubated at room temperature for 10 min in the dark. The intensity of the pink color of the samples was measured at 540 nm using a microplate reader (Bio-Rad Laboratories, Inc.). The amount of nitrite in the samples was determined using the linear sodium nitrite calibration curves at a concentration range of 2.5–100 *μ*M.

Proinflammatory cytokine levels (IL-6 and TNF-*α*) were quantified by Mouse IL-6 or Mouse TNF-*α* DuoSet® ELISA Kits (R&D systems Inc., Minneapolis, USA) according to the manufacturer's instructions. Optical density was measured at 450 nm with a microplate reader (Benchmark Plus, Bio-Rad, Japan). By preparing the standards of known cytokine concentrations, the number of cytokines in the samples was quantified from a standard curve.

### 2.11. Haematoxylin Staining

The macrophage cells were activated by LPS plus IFN-*γ* for 24 h and then stained with a haematoxylin solution to observe the phenotype feature as described by Dunkhunthod et al. [13] with some modification. Shortly, the cells were washed with PBS twice and fixed with 10% formaldehyde in PBS for 1 h. Then, cells were washed with distilled water twice and stained with haematoxylin solution for 10 min at room temperature. The stained cells were visualized under the inverted fluorescence microscope (Olympus Corporation, Japan).

### 2.12. FTIR Measurement Using Synchrotron IR Source

The sample was collected and dropped onto a barium fluoride (BaF_2_) optical window (Crystran, Crystran Ltd.) as previously described by Dunkhunthod et al. [13]. FTIR experiments were conducted using a spectroscopy facility at the Synchrotron Light Research Institute (Public Organization), Thailand. FTIR spectra were acquired in transmission mode with a Vertex 70 FTIR spectrometer coupled with a Bruker Hyperion 2000 microscope (Bruker Optics Inc., Ettlingen, Germany), using synchrotron radiation as an IR source. The microscope was equipped with 64×64 element MCT, FPA detector, which allowed simultaneous spectral data acquisition with a 36 × objective. FTIR spectrum was recorded within a spectral range of 4000–600 cm^−1^ using an aperture size of 10 *μ*m × 10 *μ*m with a spectral resolution 4 cm^−1^, with 64 scans being coadded. OPUS software (Bruker Optics Ltd., Ettlingen, Germany) was used for spectral measurement and instrument control.

The preprocessing of the spectra was performed by second derivative transformations using the Savitzky-Golay algorithm (nine smoothing points) and normalized with extended multiplicative signal correction (EMSC) using the spectral regions from 3000 to 2800 cm^−1^ and 1800 to 1400 cm^−1^. A principal component analysis (PCA) was performed using the Unscrambler® 10.5 software packages (CAMO Software AS., Oslo, Norway). Score plots (3D) and loading plots were used to represent the different classes of data and relations among variables of the data set, respectively. The integrated peak areas of FTIR spectra were analyzed using OPUS 7.2 software (Bruker) in the lipid regions (3000–2800 cm^−1^) and protein regions (1800–1400 cm^−1^) and demonstrated on a histogram.

### 2.13. Statistical Analysis

All data are expressed as the means ± SD from at least three independent experiments. The statistical significance (Statistical Package for the Social Sciences, version 19) was determined by performing a one-way analysis of variance (ANOVA) with Tukey's post hoc analysis to determine the differences among each treated group. Statistical significance was considered at *p* < 0.05.

## 3. Results and Discussion

### 3.1. GC/MS Analysis of Volatile Oils Obtained from *O. indicum*

GC/MS analysis of *O. indicum* enabled the identification of 27 volatile compounds, as shown in Table 1. The data in Table 1 illustrate retention time, chemical formula, and the relative amount of each component detected in *O. indicum*. Based on abundance, the top five major compounds present in *O. indicum* were *γ*-sitosterol (17.19%), 2-cyclohexen-1-one, 2-methyl- (15.28%), benzeneethanol, 4-hydroxy- (13.33%), 3-hydroxy-2-methylbenzaldehyde (11.18%), and cyclobutanecarboxylic acid, decyl ester (8.82%). These compounds are known to exhibit important pharmacological activity, such as antidiabetic, antioxidant, anticancer, and anti-inflammatory activities. The sterol compounds, namely, stigmasterol and *β*-sitosterol, isolated from the methanol extract of *Achillea ageratum* have been shown to possess anti-inflammatory activity against 12-0-tetradecanoylphorbol acetate- (TPA-) induced mouse ear edema [25]. The presence of phytosterols in *O. indicum* is, therefore, considered to be of great importance for the curing of diseases.

### 3.2. LC-MS/MS Analysis of Selected Flavonoids in *O. indicum*

**Figure 1:**
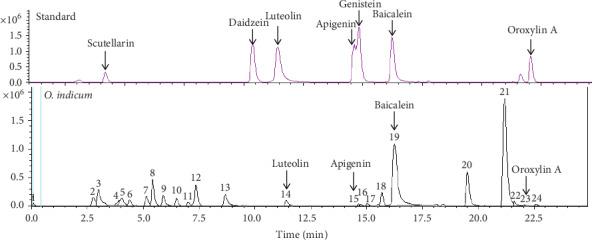
LC-MS chromatograms of *O. indicum* and standard compounds (scutellarin, daidzein, luteolin, apigenin, naringenin, genistein, baicalein, and oroxylin (A)).

The LC-MS chromatograms obtained from *O. indicum* are shown in Figure 1. Corresponding standards of scutellarin, daidzein, luteolin, apigenin, genistein, baicalein, and oroxylin A were used to identify and quantify the flavonoids composition in *O. indicum*. The predominant compounds were identified in *O. indicum*, namely, luteolin (peaks 14, RT 11.4 min) at *m*/*z* = 285, apigenin (peaks 16, RT 14.6 min) at *m*/*z* = 269, baicalein (peaks 19, RT 16.2 min) at *m*/*z* = 269, and oroxylin A (peaks 23, RT 22.0 min) m/*z* = 283. The identified compounds were quantified by comparisons of their retention time to known amounts of authentic standard. The largest amount of baicalein was detected in *O. indicum* with a concentration of 25,498.16 *μ*g g^−1^ while oroxylin A, luteolin, and apigenin were estimated in *O. indicum* at the level of 266.70, 209.98, and 77.54 *μ*g g^−1^, respectively. The previous investigation reported that *O. indicum* also contained quercetin as another flavonoid [14]. Many studies have shown high biological and pharmacological activity of flavonoid compounds. Baicalein, oroxylin, luteolin, apigenin, and quercetin, the flavonoid compounds, which were found in *O. indicum*, contributed to the antiadipogenesis, anticancer, antioxidant, and anti-inflammatory proprieties of plants [26].

### 3.3. Free Radical Scavenging and Antioxidant Activities of *O. indicum*

The antioxidative potential of *O. indicum* was assessed *in vitro* by DPPH and FRAP assays. The obtained results are shown in Table 2. In this study, the free radical scavenging activity of *O. indicum* and standard compound, VIT.C and Trolox, were determined using the DPPH-based method. The results showed that the DPPH scavenging ability of *O. indicum* and standard compound, VIT.C, were comparable and both significantly stronger than Trolox (*p* < 0.05). The ferric reducing antioxidant power (FRAP) was used to assess whether *O. indicum* had an electron-donating capacity. *O. indicum* exhibited a degree of electron-donating capacity by 57.14 ± 4.39 *μ*gVCEA mg^−1^ and 65.77 ± 4.99 *μ*gTREA mg^−1^ of dry extract. These results indicate that *O. indicum* displays an antioxidant activity based on the reducing ability to reduce ferric ion (Fe^3+^) to ferrous ion (Fe^2+^). Therefore, it could be concluded that *O. indicum* displays strong antioxidant activity in the assay used in this study. This finding is in accordance with several studies that the antioxidant activity of *O. indicum* is caused by scavenging free radical DPPH and ferric reducing antioxidant power (FRAP) [7,27,28].

### 3.4. Effects of *O. indicum* on Cell Viability in RAW264.7 Cells

**Figure 2:**
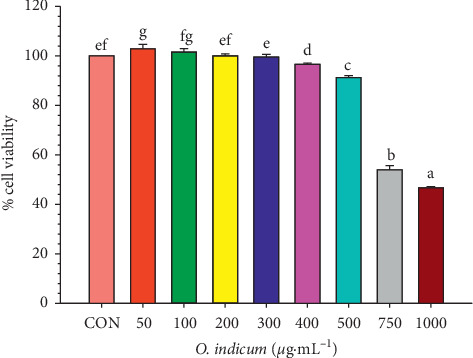
Effects of *O. indicum* on cell viability in RAW264.7 cells. Cells were treated with different concentrations of *O. indicum* for 24 h. Cell viability was determined by the MTT assay. CON: cells without *O. indicum*; 50–1,000: cells were treated with *O. indicum* at the concentration range of 50–1,000 *μ*g mL^−1^. Values are expressed as a percentage of the control. The data represent the mean ± SD of three independent experiments. Bars marked with different letters are significantly different at *p* < 0.05 as determined by one-way ANOVA with Tukey's post hoc test.

The effects of *O. indicum* on cell viability in RAW264.7 cells were comprehensively investigated, as shown in Figure 2. The cell viability of RAW264.7 cells treated with *O. indicum* at a concentration range of 50–1,000 *μ*g mL^−1^ was evaluated by using MTT assay. The viability of RAW264.7 cells was not affected by *O. indicum* when the concentrations of *O. indicum* were not greater than 300 *μ*g mL^−1^ (*p* > 0.05). This result suggests further investigation to proceed with *O. indicum* at concentrations of 50, 100, and 200 *μ*g mL^−1^ in all subsequent experiments.

### 3.5. Effects of *O. indicum* on Intracellular ROS Production in LPS plus IFN-*γ*-Activated RAW264.7 Cells

In the event of the inflammatory response, the classically activated macrophages respond to intracellular pathogens by secreting proinflammatory cytokines, chemokines, proteases, and the production of reactive oxygen species [29]. These factors are key signaling molecules that play a significant role in host defense and inflammation. The overproduction of ROS can prompt injury issues that might initiate the inflammation process [30]. Ribeiro et al. reported that flavonoid compounds possessed anti-inflammatory activity by the modulation of ROS generated through the neutrophils' oxidative burst [31].

**Figure 3:**
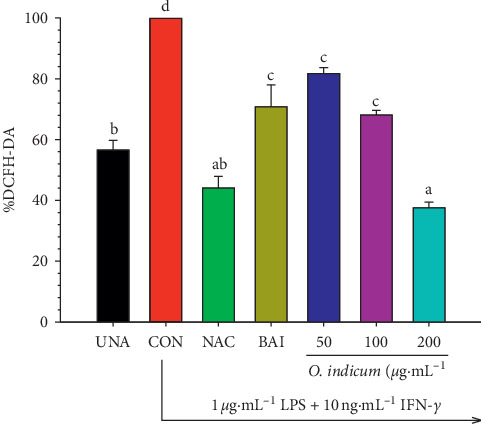
Effects of *O. indicum* on the intracellular ROS production in LPS plus IFN-*γ*-activated RAW264.7 cells. Cells were pretreated with different concentrations of *O. indicum* for 3 h and then activated with LPS plus IFN-*γ* for 24 h. Cell viability and ROS intensity are expressed as a percentage of the control. UNA: unactivated cells; CON: cells without *O. indicum*; NAC: cells were pretreated with NAC at 3 mM; VIT.C: cells were pretreated with VIT.C at 50 *μ*g mL^−1^; and 50, 100, and 200: cells were pretreated with *O. indicum* at 50, 100, and 200 *μ*g mL^−1^, respectively. Values are expressed as a percentage of the control. The data represent the mean ± SD of three independent experiments. Bars marked with different letters are significantly different at *p* < 0.05 as determined by one-way ANOVA with Tukey's post hoc test.

Therefore, a flavonoid-enriched extract from *O. indicum* undoubtedly contributes to their anti-inflammatory roles by scavenging intracellular ROS and thus be useful for preventing the uncontrolled inflammation process. To investigate whether the protective effects of *O. indicum* on the LPS plus IFN-*γ*-induced inflammatory response were due to a blockade of oxidative stress, the intracellular ROS scavenging potential of *O. indicum* was evaluated in LPS plus IFN-*γ*-activated RAW264.7 cells. As presented in Figure 3, the treatment of RAW264.7 cells with LPS plus IFN-*γ* increased ROS accumulation by 1.79-fold compared to unactivated RAW264.7 cells whereas the pretreatment with *O. indicum* significantly inhibited the ROS generation in a dose-dependent manner. Compared to LPS plus IFN-*γ*-activated RAW264.7 cells, *O. indicum* at a concentration of 50, 100, and 200 *μ*g mL^−1^ reduced intracellular ROS accumulation to 81.08 ± 3.44, 68.16 ± 3.34, and 36.35 ± 1.62%, respectively. The inhibitory effects of *O. indicum* on ROS accumulation at 35% (IC_35_), 40% (IC_40_), and 50% (IC_50_) were determined to be 106.03 ± 5.71, 122.72 ± 4.94, and 156.10 ± 4.36 *μ*g mL^−1^, respectively. Surprisingly, the intracellular ROS scavenging activity of *O. indicum* (IC_50_, 156.10 ± 4.36 *μ*g mL^−1^) is, therefore, approximately 3 times more effective than a selective ROS scavenger, NAC (IC_50_, 3 mM or 489.57 *μ*g mL^−1^). The antioxidant compound, VIT.C, inhibited intracellular ROS by 40% inhibition (IC_40_) at 50 *μ*g mL^−1^, which was 2.45 times greater than *O. indicum* (IC_40_ = 122.72 ± 4.94 *μ*g mL^−1^) when compared to the same inhibitory activity. These results suggest that *O. indicum* possesses a vigorous antioxidant activity in scavenging ROS secreted by LPS plus IFN-*γ*-stimulated in RAW264.7 cells.

Mairuae et al. demonstrated that *O. indicum* treatment attenuated the generation of ROS on A*β*25-35-induced cell injury in human neuroblastoma SH-SY5Y cells [32]. Mohan et al. found that *O. indicum* leaf extract could overcome the oxidative stress induced by 4-NQO in albino Wistar rats when administered orally [33]. These findings lead us to believe that flavonoids present in *O. indicum*, baicalein, quercetin, luteolin, and apigenin may play an important role in protection against oxidative stress.

Base on the LC-MS experiment, the results indicated that *O. indicum* at a concentration of 200 *μ*g mL^−1^ is composed of baicalein of about 5 *μ*g mL^−1^. In order to clarify whether baicalein could act as an intracellular ROS scavenger or not, baicalein 5 *μ*g mL^−1^ was used as a positive control. The result indicated that baicalein decreased LPS plus IFN-induced intracellular ROS levels by approximately 30%. These data suggest that baicalein in *O. indicum* can scavenge the ROS production of test cells.

Previous investigators demonstrated that baicalein enhanced cellular antioxidant defense capacity in C6 glial cells by inhibiting ROS production and activating the Nrf2 signaling pathway [34]. Qi et al. [35] found that baicalein reduced LPS-induced inflammation via suppressing JAK/STATs activation and ROS production. In addition, baicalein reduced H_2_O_2_-induced DNA damage as a result of a decrease in phospho-H2A.X production, DNA tail formation, and lipid peroxidation prevention [36]. Our results indicated that the pretreatment of RAW264.7 cells with *O. indicum* at a concentration of 200 *μ*g mL^−1^ significantly decreased the intracellular ROS accumulation by approximately 64%. This finding indicates that *O. indicum* at 200 *μ*g mL^−1^, which contains baicalein around 5 *μ*g mL^−1^, shows significantly stronger radical scavenging potency than baicalein alone (*p* < 0.05, Figure 3). The higher potency of *O. indicum* may be due to the other bioactive compounds present in *O. indicum* such as *γ*-sitosterol, stigmasterol, luteolin, apigenin, and quercetin, which could act as ROS scavenging agents. All compounds above have been shown to possess antioxidant properties. The intracellular ROS scavenging potency of *O. indicum* is more than two times stronger compared to baicalein alone which leads us to believe that synergistic activity could take place between baicalein and other flavonoids or volatile compounds [37].

### 3.6. Effects of *O. indicum* on Nitric Oxide (NO) and Proinflammatory Cytokine (IL-6 and TNF-*α*) Production in LPS plus IFN-*γ*-Activated RAW264.7 Cells

During inflammation, the macrophages actively participate in the inflammatory response by releasing cytokines (TNF-*α*, IL-1*β*, and IL-6), chemokines, and inflammatory mediators (NO, iNOS, PGE_2_, and COX-2) [38]. The overproduction of these agents contributes to the induction and progression of several inflammatory diseases. Thus, it is crucial to regulate the inflammatory mediators in controlling the inflammatory progression and treating inflammatory disorders.

Nitric oxide (NO) is synthesized by many cell types involved in immunity and inflammation. NO is vital as a toxic defense molecule against infectious organisms. On the other hand, NO reacts rapidly with superoxide to form the more reactive product, peroxynitrite (ONOO^−^), which can directly react with various biological targets and components of the cell, including lipids, thiols, amino acid residues, DNA bases, and low-molecular-weight antioxidants [39].

**Figure 4:**
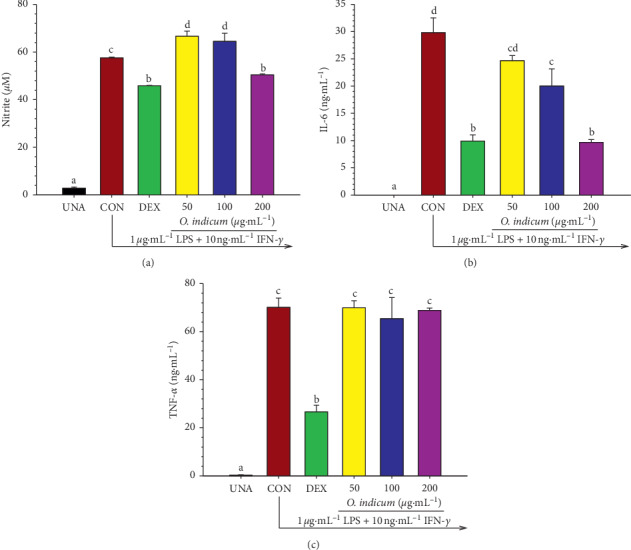
Effects of *O. indicum* on (a) NO production, (b) proinflammatory cytokines IL-6, and (c) TNF-*α* secretion in LPS plus IFN-*γ*-activated RAW264.7 cells. Cells were pretreated with different concentrations of *O. indicum* for 3 h and then activated with LPS plus IFN-*γ* for 24 h UNA: unactivated cells; CON: cells without *O. indicum*; DEX: cells were pretreated with DEX at 1 *μ*M; 50, 100, and 200: cells were pretreated with *O. indicum* at 50, 100, and 200 *μ*g mL^−1^, respectively. The data represent the mean ± SD of three independent experiments. Bars marked with different letters are significantly different at *p* < 0.05 as determined by one-way ANOVA with Tukey's post hoc test.

Therefore, the anti-inflammatory potential of *O. indicum* on inhibition of NO production was measured after the treatment of *O. indicum* in LPS plus IFN-*γ*-activated RAW264.7 cells. The anti-inflammatory agent, DEX, was selected to serve as the reference drug. As shown in Figure 4(a), upon LPS plus IFN-*γ* treatment (CON), NO production was increased with the nitrite level peaking to 54.17 ± 0.38 *μ*M. However, pretreatment of cells with the highest concentration (200 *μ*g mL^−1^) of *O. indicum* suppressed the production of NO of about 16%, which is precisely the same efficiency as 1 *μ*M DEX, compared to the LPS plus IFN-*γ*-activated group.

Qi et al. reported that baicalein suppressed LPS-induced inflammatory responses in RAW264.7 macrophages via attenuating NO synthesis [35]. Shimizu et al. noted that an equimolar mixture (F-mix) of baicalein, wogonin, and oroxylin A showed a synergistic inhibitory effect on the production of NO in LPS-treated J774.1 cells [40]. Also, a mixture of *β*-sitosterol and stigmasterol isolated from *Andrographis paniculata* significantly suppressed NO production in LPS/IFN-*γ* stimulated RAW264.7 cells [41].

Both IL-6 and TNF-*α* are essential proinflammatory cytokines, either of which can serve as an indicator of inflammation. To confirm the anti-inflammatory effect of *O. indicum,* we also investigated the inhibitory effect of *O. indicum* on proinflammatory cytokine secretion by measuring IL-6 and TNF-*α* levels in RAW264.7 cells activated by LPS plus TNF- *α*. Exposure of the cells with LPS plus IFN-*γ* strongly activated the secretion of IL-6 and TNF- *α* compared to the unactivated group (Figures 4(b) and 4(c)) [42]. The results showed that DEX markedly reduced the secretion of IL-6 and TNF-*α* of about 69.34% and 62.02%, respectively, compared to the LPS plus IFN-*γ*-activated group. Treatment with *O. indicum* inhibited the secretion of IL-6 in a dose-dependent manner (Figure 4(b)), but it did not affect the reduction of TNF-*α* level (*p* > 0.05, Figure 4(c)). The concentration of *O. indicum* at 200 *μ*g mL^−1^ exerted an IL-6 inhibition by 62.99%, which was practically similar to the reference drug, DEX (69.34% inhibition).

Other researchers had demonstrated that the ethyl acetate extract derived from the stem bark of *O. indicum* showed the inhibitory effect on LPS-induced IL-6, IL-1*β*, and TNF-*α* release in human monocytes [7]. *In vivo* study indicated that the aqueous decoction of both stem bark and root bark of *O. indicum* produced anti-inflammatory activity by reducing paw edema formation in the carrageenan-induced paw edema model [42]. Furthermore, flavonoids found in this plant, such as baicalein, apigenin, oroxylin A, and luteolin, are known for their anti-inflammatory effects attributed at least partially through the suppression of proinflammatory cytokines, IL-6, IL-1*β*, and TNF-*α* [43]. Moreover, it had been reported that a mixture of *β*-sitosterol and stigmasterol isolated from *Andrographis paniculata* significantly decreased TNF-*α* and IL-6 secretion from LPS/IFN-*γ*-stimulated RAW264.7 cells [41]. These findings provide evidence that the main constituents of *O. indicum* are flavonoids and phytosterols, which could potentially act as the anti-inflammatory compounds of this plant.

Differential effects of *O. indicum* on IL-6 and TNF-*α* production in RAW264.7 cells could be explained by the different mechanisms of *O. indicum* on the secretion of IL-6 and those of TNF-*α* alleviation. Several studies reported that the mechanism, signaling pathways, and transcription factors controlling TNF-*α* expression were distinct from those of IL-6 [44–46]. It had been reported that the activation of p38 MAPK was required for the LPS/TLR4-induced expression of TNF-*α*, but not IL-6 [44]. It is well known that CREB recognizes a similar DNA binding sequence in the promoter region of the TNF-*α* gene [47]. In contrast, similar sequences have not been identified on the IL-6 and IL-1 promoters. Also, the STAT3 tyrosine phosphorylation is a key role to induce IL-6 production in response to inflammation. *In vitro* study revealed that the blocking STAT3 activity preferred to inhibit LPS-mediated production of IL-1*β* and IL-6, but not TNF-*α*, in RAW264.7 cells [46]. The study of Prêle et al. also confirmed that the STAT3 activation did not directly regulate LPS-induced TNF-*α* production in human monocytes [48].

### 3.7. Effects of *O. indicum* on the Morphology of LPS plus IFN-*γ*-Activated RAW264.7 Cells

**Figure 5:**
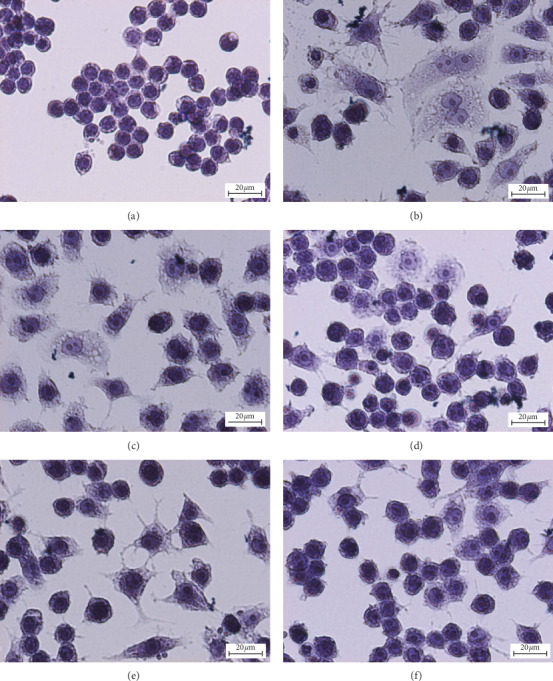
Effects of *O. indicum* on the morphology of LPS plus IFN-*γ*-activated RAW264.7 cells. Haematoxylin staining of 6 different groups of the sample. a: unactivated RAW264.7 cells; b: activated RAW264.7 cells (cells without *O. indicum*); c: cells were pretreated with DEX at 1 *μ*M; d, e, and f: cells were pretreated with *O. indicum* at 50, 100, and 200 *μ*g mL^−1^, respectively (original magnification at ×400, scale bar; 20 *μ*m).

RAW264.7 cells were pretreated with different concentrations of *O. indicum* for 3 h and then activated by LPS plus IFN-*γ* for another 24 h. Morphology change of RAW264.7 cells was observed under microscopy, as shown by haematoxylin staining (Figure 5). The unactivated RAW264.7 cells showed the round morphology, whereas LPS plus IFN-*γ*-activated RAW264.7 cells showed enlargement, dendritic, spindle, and spheres, which were the phenotype features of activated macrophages [49]. The previous study indicated that the morphological changes of adherent macrophages were associated with their secretion of inflammatory cytokines [50]. These cells treated with *O. indicum* at 200 *μ*g mL^−1^ displayed less activated macrophage phenotypes than activated RAW264.7 cells, DEX- and *O. indicum* treated at 50 and 100 *μ*g mL^−1^.

### 3.8. Effects of *O. indicum* on Biomolecular Changing Detected by SR-FTIR

We found that the extract of *O. indicum* possessed antioxidant potential by scavenging intracellular ROS, anti-inflammatory activity, and suppressing proinflammatory mediators (NO) and cytokine secretion (IL-6) in LPS plus IFN-*γ*-activated RAW264.7 cells. The SR-FTIR investigation was conducted to determine the effect of *O. indicum* on the cellular biochemical alterations in LPS plus IFN-*γ*-activated RAW264.7 cells.

**Figure 6:**
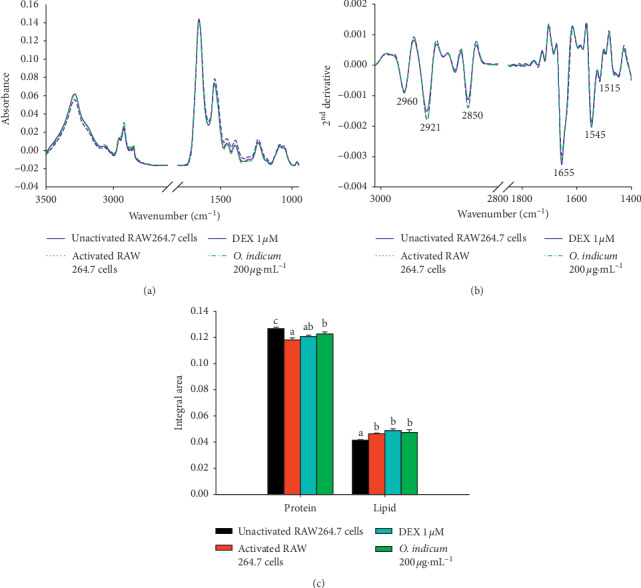
Effects of *O. indicum* on biomolecular changes detected by FTIR. (a) Average original FTIR spectra (3500–950 cm^−1^). (b) Average the secondary derivative spectra of lipid regions (3000–2800 cm^−1^) and protein regions (1800–1400 cm^−1^). (c) The bar graph of integrated areas of lipid regions (3000–2800 cm^−1^) and protein regions (1800–1400 cm^−1^). The data obtained from unactivated RAW264.7 cells (*n* = 120), activated RAW264.7 (LPS plus IFN-*γ*) (*n* = 120), and activated RAW264.7 (LPS plus IFN-*γ*) exposed to 1 *μ*M DEX (*n* = 157) or 200 *μ*g mL^−1^ of *O. indicum* (*n* = 135). Data are represented as means ± SD for three replicates. Bars marked with different letters are significantly different at *p* < 0.05 as determined by one-way ANOVA with Tukey's post hoc test.

The average original FTIR spectrum bands of unactivated RAW264.7 cells, activated RAW264.7 cells, and activated RAW264.7 cells treated with *O. indicum* (200 *μ*g mL^−1^) or DEX (1 *μ*M) are displayed in Figure 6(a). The raw spectrum was more useful to perform the second derivative analysis in spectral regions ranging from 3,000 to 2800 cm^−1^ for lipid regions and from 1800 to 1400 cm^−1^ for protein regions, as shown in Figure 6(b). The signal intensity and area of the peaks of protein and lipid regions of these groups are calculated and shown in Figure 6(c). Moreover, the FTIR band assignments are shown in Table 3.

The change in cellular lipid was observed in the mainly lipid region (3000–2800 cm^−1^). The average second derivative spectra of RAW264.7 cells under different experimental conditions exhibited three specific regions at 2960 cm^−1^, 2921 cm^−1^, and 2850 cm^−1^, which are assigned to the asymmetrical stretching vibrations of the CH_3_ and CH_2_ groups of the phospholipids membrane and CH_2_ symmetric stretching, respectively (Figure 6(b)). The relative absorbance at 2960 cm^−1^, 2921 cm^−1^, and 2850 cm^−1^ in activated RAW264.7 cells, DEX-, or *O. indicum*-treated activated RAW264.7 cells was higher than that in the unactivated RAW264.7 cells. The integrated areas of lipid regions of the second derivative spectra were calculated for unactivated and treated activated RAW264.7 cells in order to quantify the lipid content [49]. The results exhibited that the integrated area of the lipid region was significantly increased in activated RAW264.7 cells, DEX-, and *O. indicum*-treated activated RAW264.7 cells compared to the unactivated RAW264.7 cells (*p* < 0.05). The increase of lipid regions in LPS plus IFN-*γ*-activated RAW264.7 cells under three different experimental conditions could be related to cell membrane changes. The changes in the membrane are due to phenotype features of activated macrophage, including enlargement, dendritic, and spherical features (Figure 5), which developed in response to the inflammatory inducer during inflammation [49]. Moreover, these results are consistent with Funk et al.'s study [51] showing that the activation of RAW264.7 macrophages enhances their ability to accumulate lipid from a variety of lipid molecules and to become foam cells.

The cellular protein change was observed within 1800 cm^−1^ and 1400 cm^−1^ interval, reflecting the vibrations of the amide I (1700–1600 cm^−1^) and amide II (1600–1400 cm^−1^) (Figure 6(b)). In unactivated RAW264.7 cells, a strong peak at 1655 cm^−1^ and 1545 cm^−1^ assigned to stretching vibrations of *α*-helix secondary protein structure displayed higher signal intensity than other groups. These results are in accordance with the integrated areas of the protein regions where the protein content in unactivated RAW264.7 cells is significantly greater than that in the activated RAW264.7 cells and DEX- or *O. indicum*-treated activated RAW264.7cells (*p* < 0.05, Figure 6(c)). Thus, these results reflect the altered cellular protein profile in all treated groups of LPS plus IFN-*γ*-activated RAW264.7 cells compared to unactivated RAW264.7 cells. Upon treatment of cells with *O. indicum*, the protein content was significantly increased compared to LPS plus IFN-*γ*-activated RAW264.7 cells (*p* < 0.05). These results suggest that *O. indicum* treatment could protect the molecule of proteins, probably because of their antioxidant potential. Excessive production of reactive oxygen species is frequently observed during the inflammatory response, causing the oxidation of proteins, lipid peroxidation, nucleic acid destruction, and enzyme inhibition [52]. Accordingly, the oxidized proteins thus become better targets for proteolytic digestion by the 20S proteasomes and, consequently, decrease the levels of the proteins in general [53]. Flavonoids play an important role as a ROS scavenger by locating and neutralizing radicals before they damage the cell structure [54]. Therefore, the flavonoid-enriched extract from *O. indicum* could protect proteins from oxidation under oxidative stress, and proteolytic digestion leads to an increase in cellular protein contents.

However, there was no significant difference between the FTIR spectra of unactivated RAW264.7 cells and other groups in nucleic acid regions (1,300–900 cm^–1^) (data are not shown). The data indicated that the selected concentration of *O. indicum* did not produce any effect on nucleic acids (DNA and RNA base) of the cells, which was consistent with its cytotoxicity evaluation by using MTT assay. Supporting our results, Zelig et al. reported that a decrease in DNA absorbance was associated with apoptotic cell death, by contrast, to increase during necrotic cell death [55].

**Figure 7:**
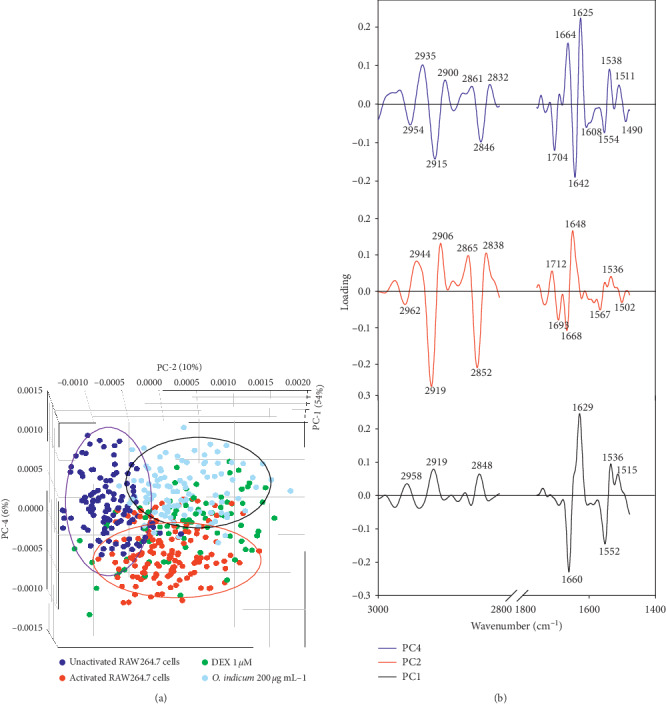
PCA analysis of FTIR spectral range 3000–2800 cm^−1^ and 1800–1400 cm^−1^ giving (a) PCA 3D score plot and (b) PCA loading plot. PCA score plots showed distinct clustering between unactivated RAW264.7 cells (*n* = 120), activated RAW264.7 (LPS plus IFN-*γ*) (*n* = 120), and activated RAW264.7 (LPS plus IFN-*γ*) exposed to 1 *μ*M DEX (*n* = 157) or 200 *μ*g mL^−1^ of *O. indicum* (*n* = 135). PCA loading plots identify biomarker differences over a spectral range of samples.

In order to discriminate the distinct clustering of spectra from the four cell populations, PCA analysis was performed to identify which wavenumbers in the FTIR spectra complex showed the largest spectral variation within the sample. The 3-dimensional PCA clustering results from FTIR spectral data of unactivated RAW264.7 cells, activated RAW264.7 cells, and activated RAW264.7 cells after treatment with DEX or *O. indicum* are displayed in Figure 7(a). The PCA score plot demonstrated that the clusters of unactivated RAW264.7 cells and *O. indicum*-treated activated RAW264.7 cells were separated from activated RAW264.7 cells and DEX-treated activated RAW264.7 cells along PC1 (54%) and PC4 (6%) whereas the clusters of unactivated RAW264.7 cells were distinguished from *O. indicum*-treated activated RAW264.7 cells along PC2 (10%). The PCA loading plots (Figure 7(b)) were used to identify the regions of the spectrum, which most contributed to the clustering. The positive score of the spectra of the unactivated RAW264.7 cells and *O. indicum*-treated activated RAW264.7 cells was clearly separated from the negative score of the spectra of the other two groups along with PC1 score plot, which displayed remarkably high negative PC1 loadings at 1660 cm^−1^ and 1552 cm^−1^ (suggesting an *α*-helix protein structure of amide I and amide II, respectively). PC2 loading plot was discriminated by the negative loading spectra at 2919 cm^−1^ and 2852 cm^−1^ caused by the C-H stretching assigned to the lipids and at 1693 cm^−1^ and 1668 cm^−1^ resulting from the *α*-helix protein structure of amide I, which separated the positive score of DEX-treated activated RAW264.7 cells from the negative score of the unactivated RAW264.7 cells. The amide I band from proteins at 1642 cm^−1^ (assigned to the *α*-helix structure) and the C-H stretching region (assigned to the lipids) were heavily loaded for PC4 which separated the positive score of the spectra of the unactivated RAW264.7 cells and *O. indicum*-treated activated RAW264.7 cells from the negative score of the spectra of the activated RAW264.7 cells and DEX-treated activated RAW264.7 cells. These results are consistent with its second derivative spectra and the integrated areas of protein and lipid regions.

## 4. Conclusions

In conclusion, these findings provide evidence that *O. indicum* could possess the antioxidant and anti-inflammatory effects in LPS plus IFN-*γ*-activated RAW264.7 cells by scavenging intracellular ROS, reducing NO and IL-6 secretion, respectively. These pharmacological properties of *O. indicum* may occur from the synergistic interaction between its flavonoids and phytosterol components. These results also provide the first evidence of the potential use of SR-FTIR microspectroscopy to evaluate the biochemical profile alteration of activated RAW264.7 macrophages. Therefore, *O. indicum* may be used as a potential source of nutraceutical for the development of health food supplement or a novel anti-inflammatory herbal medicine to alleviate the excessive inflammatory response.

## Figures and Tables

**Table 1:** GC-MS analysis of *O. indicum.*

No.	Compound name	Formula	RT	%area
1	2-Furancarboxaldehyde, 5-(hydroxymethyl)-	C_6_H_6_O_3_	4.88	4.86
2	Nonanoic acid	C_9_H_18_O_2_	5.30	2.28
3	n-Decanoic acid	C_10_H_20_O_2_	6.55	2.82
4	2-Cyclohexen-1-one, 2-methyl-	C_7_H_10_O	7.13	15.28
5	2-Dodecenoic acid	C_12_H_22_O_2_	7.30	1.33
6	Benzeneethanol, 4-hydroxy-	C_8_H_10_O_2_	7.54	13.33
7	3-Hydroxy-2-methylbenzaldehyde	C_8_H_8_O_2_	7.88	11.18
8	Cyclobutanecarboxylic acid, decyl ester	C_17_H_28_O_2_	8.89	8.82
9	Dodecanoic acid	C_12_H_24_O_2_	8.98	1.15
10	Ethyl N-(o-anisyl)formimidate	C_10_H_13_NO_2_	9.59	0.40
11	1,6-Dihydro-5-(2-hydroxyethyl)-4-methyl-6-oxopyrimidine	C_7_H_10_N_2_O_2_	10.99	1.39
12	Tetradecanoic acid	C_14_H_28_O_2_	11.26	0.21
13	Hexadecanoic acid, methyl ester	C_17_H_34_O_2_	13.58	0.28
14	n-Hexadecanoic acid	C_16_H_32_O_2_	14.28	0.66
15	Hexadecanoic acid, ethyl ester	C_18_H_36_O_2_	14.82	0.99
16	Phytol	C_20_H_40_O	17.09	0.39
17	Linoleic acid ethyl ester	C_20_H_36_O_2_	17.95	0.58
18	Linolenic acid ethyl ester	C_20_H_34_O_2_	18.08	0.57
19	Glycerol 1,3-dipalmitate	C_35_H_68_O_5_	20.36	1.13
20	Linolelaidic acid, methyl ester	C_19_H_34_O_2_	23.30	0.79
21	9,12,15-Octadecatrienoic acid, 2-phenyl-1,3-dioxan-5-yl ester	C_28_H_42_O_4_	23.44	0.52
22	Dotriacontane	C_32_H_66_	23.67	1.29
23	Glycerol 1-monopalmitate	C_19_H_38_O_4_	23.93	4.37
24	*β*-Monolinolein	C_21_H_38_O_4_	26.70	4.09
25	Campesterol	C_28_H_48_O	35.57	2.48
26	Stigmasterol	C_29_H_48_O	36.33	1.48
27	*γ*-Sitosterol	C_29_H_52_O_2_	37.88	17.19

**Table 2:** Total antioxidant (FRAP) and DPPH scavenging activities of *O. indicum* and standard compounds.

Sample	FRAP values		DPPH scavenging activity (IC_50_) *μ*g mL^−1^
	(*μ*gVCEA mg^−1^)	(*μ*gTREA mg^−1^)	
*O. indicum*	57.14 ± 4.39	65.77 ± 4.99	43.28 ± 0.67^a^
VIT.C	—	—	44.57 ± 0.59^a^
Trolox	—	—	67.19 ± 4.82^b^

Values are mean ± SD (*n* = 3) and are representative of three independent experiments with similar results. Different letters within the same column are significantly different at *p* < 0.05.

**Table 3:** FTIR band assignments.

Band position of 2^nd^ derivative spectra (cm^−1^)	Assignments
2960	CH_3_ stretch (antisymmetric) due to methyl terminal of membrane phospholipids
2921	CH_2_ antisymmetric stretch of methylene group of membrane phospholipids
2850	CH_2_ symmetric stretching: mainly lipids
1655	Amide I: C=O (80%) and C–N (10%) stretching, N–H (10%) bending vibrations: Proteins *α*-helix
1545	Amide II: N–H (60%) bending and C–N (40%) stretching vibrations: proteins *α*-helix

## Data Availability

The datasets used and analyzed during this study are available from the corresponding author upon reasonable request.
